# Physiotherapy for epidermolysis bullosa: clinical practice guidelines

**DOI:** 10.1186/s13023-021-01997-w

**Published:** 2021-09-30

**Authors:** Amy Weisman, Jennifer M. Chan, Chantal LaPointe, Kaye Sjoholm, Kristy Steinau, Kaycie Artus, Suci Widhiati, Rebecca Bodan, Michelle Wood, Julio C. Salas-Alanis, Anna Carolina Rocha, Beata Faitli, Phuong Khuu

**Affiliations:** 1 Department of Rehabilitation Services, Stanford Children’s Health, 321 Middlefield Road, Menlo Park, CA 94025 USA; 2 Physiotherapy, Centre Hospitalier Universitaire Sainte-Justine, Montreal, Canada; 3 Department of Rehabilitation, Phoenix Children’s Hospital, Phoenix, AZ USA; 4 Cincinnati Children’s Hospital Medical Center, Cincinnati, OH USA; 5 Camp Spirit, EB Winter Camp in Colorado, Golden, CO USA; 6 Pediatric Dermatology Division, Department of Dermatology and Venereology, Faculty of Medicine, Universitas Sebelas Maret - Dr. Moewardi General Hospital, Surakarta, Indonesia; 7 School of Nursing, California State University Fullerton, Fullerton, CA USA; 8 Department of Physiotherapy, Great Ormond Street NHS Foundation Trust, London, UK; 9 Dermatology Department, Instituto de Dermatologia Jalisco, Guadalajara, Jalisco Mexico; 10 DEBRA Mexico, Monterrey , Nuevo Leon Mexico; 11 DEBRA Brazil, Blumenau, Santa Catarina Brazil

**Keywords:** Physiotherapy, Physical therapist, Epidermolysis bullosa, Mobility, Weight bearing, Motor development, Clinical practice guideline

## Abstract

Epidermolysis bullosa (EB) is characterized by skin fragility with blister formation occurring spontaneously or following minor trauma such as gentle pressure or friction. Current physiotherapy practice is based on anecdotal care, clinical expertise and creative problem solving with caregivers and individuals with EB. Evidence based intervention is needed to establish a foundation of knowledge and to guide international practitioners to create and improve standards of care to effectively work with individuals living with EB. This clinical practice guideline (CPG) was created for the purpose of providing evidence based interventions and best clinical practices for the physiotherapy management of individuals with EB. A survey was conducted within the EB community and six outcomes were identified as a priority to address in physiotherapy management, including (1) attaining developmental motor milestones, (2) identifying safe and functional mobility in the natural environment, (3) encouraging ambulation endurance, (4) supporting safe ability to bear weight, (5) improving access to physiotherapy services, and (6) optimizing interaction with the community. A systematic literature review was conducted and articles were critically analyzed by an international panel consisting of thirteen members: healthcare professionals (including physiotherapist, doctors, and occupational therapist), caregivers, and individuals with EB. Recommendations were formulated from evidence and panel consensus. An external panel of twelve were invited to improve the quality and gather feedback on draft manuscript and recommendations. This CPG describes the development of recommendations for physiotherapy management including several best practice interventions. This guideline lays the foundational work for physiotherapist throughout the world to provide high quality services while improving and maintaining functional mobility and independence within the EB community. The CPG outlines limitations in the evidence available and possible future research needed to improve physiotherapy practice.

**Supplementary Information:**

The online version contains supplementary material available at 10.1186/s13023-021-01997-w.

## Introduction

Epidermolysis bullosa (EB) is a rare genetic disorder characterized by skin fragility with blister formation occurring spontaneously or following minor trauma such as gentle pressure or friction. Classification of EB is based on where the splitting occurs in the different layers of skin at a microscopic level. The four major types, subtypes and common areas affected that can lead to functional limitations are outlined in Table 1 [1].

**Table 1:** Classification of EB types and subtypes

Major EB types	EB subtypes	Main problems and common areas affected that can lead to functional limitations
EB simplex (EBS)	Localized	Blistering appears at birth or early infancy; mainly appears on hands and feet; plantar keratoderma results in pain, leading to inactivity and indirectly to obesity and dependence on mobility devices
	Intermediate	Blistering appears at birth; generalized but less severe; development of plantar keratoderma; Cardiomyopathy in EBS is a feature only seen in KLHL24 deficiency; Muscular dystrophy in EBS is a feature of EBS with plectin deficiency/PLEC mutations
	Severe	Blistering appears at birth and the skin is noticeably fragile; widespread large blisters with minimal trauma, ulcerated areas on hands and feet and the oral mucosa can also be affected; development of plantar keratoderma; neonatal complications can be life threatening in the first year of life
Junctional EB (JEB)	Intermediate	Blistering is generalized but less severe; with possible development of chronic granulation tissue; development of plantar keratoderma
	Severe	Infrequent blistering in neonatal period, though wounds can become chronic, hypergranulated, friable and persistently large; laryngeal mucosa is affected and life threatening airway obstruction can occur; dependence on respiratory therapy; death usually occurs in first 24 months of life
Dystrophic EB (DEB)	Localized Dominant DEB (DDEB)	Skin usually fragile from birth or early childhood, but limited to certain areas of fingers, nails, toes, and shins
	Intermediate DDEB	Skin fragility, scarring and milia are generalized and present from birth; mild flexion contracture can occur as can web space fusion; esophageal blistering is common and corneal erosions
	Intermediate Recessive DEB (RDEB)	More severe symptoms than DDEB; mucosal involvement, though is not very common
	Severe RDEB	Widespread blistering from birth with marked fragility; frequent occurrence of aplasia cutis, with the potential for contracture formation; progressive pseudosyndactyly at fingers and toes and flexion contractures are common, eventually leading to mitten deformities; scarring can lead to microstomia which can result in dental issues; esophageal blistering and strictures are common; anemia and inflammation are common as is osteopenia, osteoporosis and vertebral fracture; squamous cell carcinoma
Kindler EB (KEB)		Generalized blistering with a tendency to affect extremities; blistering decreases with age; squamous cell carcinoma

The severity of the condition is dependent on the pathology in relation to its types and sub-types, and the depth at which the blisters form. Considerable variations exist in disease severity and the natural history of patients, within even a single EB subtype or related pathological occurrence, because of the influence of environmental and/or modifying genetic factors. Individuals with EB do present with a wide range of disabilities; the most severely affected may have scarring, fibrosis and contractures affecting any part of the body. Acute or chronic pain and pruritus are also impairments that can limit and affect each individual person’s overall development, including, but not limited to delays in gross motor skills, limitations in functional mobility and decreased functional capacity to participate as a member of their family and in the community [2]. When movement becomes uncomfortable and painful, it can lead to restriction of joints and thus affects functional activities and ultimately everyday life. Impact of mental health and wellness can also result in loss of functional capacity and ability to actively participate in society.

There is no cure for EB, therefore supportive care for relief of symptoms should be provided by an interdisciplinary team (IDT) whenever possible. A physiotherapist is an essential member of the IDT in treating individuals with EB.

Physiotherapy is the art and science of physical or corrective rehabilitation. This practice includes the promotion and maintenance of physical fitness to enhance the bodily movement related to health and wellness of individuals through the use of physiotherapy interventions. Physiotherapists assist with:

- preventing disease, injury, and disability;
- managing acute and chronic conditions, activity limitations, and participation restrictions;
- improving and maintaining optimal functional independence and physical performance;
- rehabilitating injury and the effects of disease or disability with therapeutic exercise programs and other interventions;
- educating and planning maintenance and support programs to prevent re-occurrence, re-injury or functional decline.

Physiotherapy is anchored in movement sciences and aims to enhance or restore function of multiple body systems. They are committed to enhancing health, lifestyle and quality of life (QoL) in a holistic approach. Management commonly includes assistance with specific exercises, manual therapy, working with individuals to prevent loss of mobility, recommending assistive devices, encourage functional movement, and restoring maximum movement. Practice is typically guided by evidence-based interventions [3]. With the rare diagnosis of EB, there is limited research and resources available to physiotherapists.

Thus, current physiotherapy practices with individuals with EB are based on anecdotal care, clinical expertise and collaboration between caregiver and patient. Evidence-based clinical practice guidelines (CPGs) are needed to establish a foundation of knowledge to guide international practitioners to create and improve standards of care and to be able to work effectively with those living with the rare diagnosis of EB [2].

Objectives of this CPG were (a) to provide evidence based recommendations for the management of physiotherapy services for patients with EB, (b) to provide a CPG for any physiotherapists that might encounter a patient with EB for the first time and provide them with resources on justifying improved mobility, flexibility and strengthening activities to improve cardiovascular involvement so that patients with EB can improve their QoL and (c) to identify areas where there is lack of evidence and promote future physiotherapy research.

This guideline is aimed at physiotherapists, people with EB and their caregivers, rehabilitation practitioners, allied health professionals, nurses, occupational therapists (OTs), physicians/medical doctors, physician assistants, psychomotor therapists, nurse practitioners, social workers, educational staff and employers of individuals with EB. With intention to provide guideline recommendations to all subtypes of EB (Table 1) and all ages, we found these recommendations are more applicable to the severe recessive dystrophic EB population as there was limited research available to critically analyze for every EB subtype. Internationally, this has important implications for a physiotherapist to help enhance their proficiency in the functional treatment of people with EB, a condition requiring specialist intervention beyond just ‘being more careful’. They must avoid causing secondary injury by purposefully handling body and limbs strategically, avoid the use of highly adhesive tapes, dressings and felt padding, and by removing any adherent dressings, ideally with silicone spray. The recommendations can be generalized for people with EB and of all ages, who have the potential to experience, or are experiencing, physical limitations.

## Method

In 2017, an international panel of 13 members was coordinated by DEBRA International through a voluntary membership. They represented physiotherapists, OT, nurses, dermatologists, parent/caregiver, person with EB, as experts (Additional file 1).

A scoping survey was created and focused on topics relevant to physiotherapists. The survey was distributed to health care providers working with patients with EB, caregivers, and people living with EB in the US, UK, Australia, Spain, and Croatia. The data (n = 33) informed the first meeting to identify the clinical questions and outcomes prioritized for the CPG. The meeting was held in Salzburg, Austria during the 2017 EB CLINET conference with eight in-person panel members, three panel members via teleconference and remaining panel members provided input through minutes.

**Fig. 1:**
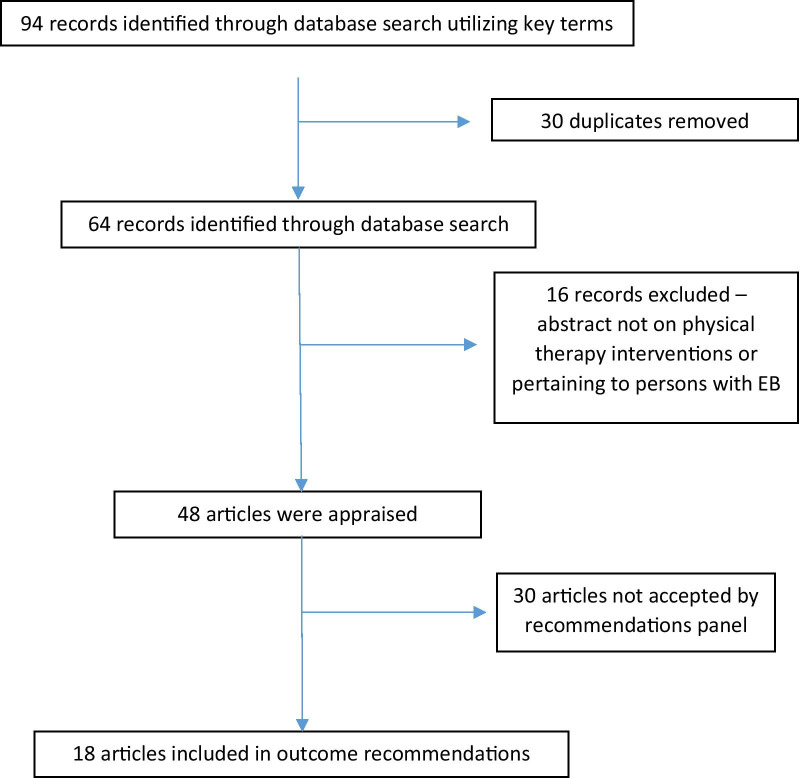
Search and filtration results

A systematic literature review using eight search engines was conducted based on key terms, including years 1990 – 2020 with no language limits (Additional file 2)., A total of 64 articles were identified and filtered by title, abstract and diagnoses for potential inclusion regardless of study design (Fig. 1). Only articles pertaining to people with EB were selected. All articles were appraised in full by at least two appraisers for consistency. 18 articles were categorized based on outcomes with some articles overlapping in more than one outcome. Summary tables of all six outcomes were presented at the final meeting in Phoenix, Arizona, July 2018 for grading the strength of the recommendations. The CPG draft was circulated to an external panel of 12, to obtain feedback on the manuscript and recommendations (Additional file 1). The external panel consisted of EB stakeholders, who signed a roles and responsibility agreement, reported no conflicts of interest and volunteered through DEBRA International CPG Network or EB-CLINET. The Appraisal of Guidelines for Research and Evaluation (AGREE) [4] II tool was consulted to increase the quality of practice guidelines in rare diseases. The panel addressed all reviewer comments in the final editing stage.

## Recommendations

The key recommendations with levels of evidence and grades of this guideline are summarized in Table 2. Detailed recommendations are outlined below.

**Table 2:** Summary of recommendations by outcome

	Strength of recommendation	Quality of evidence	Key references
A. Desirable consequences *probably outweigh* undesirable consequences in most settings, for this reason we suggest offering these options			
**R1. Optimize developmental motor milestone attainment**	**D**		
Aim of physiotherapy is to prevent contractures and reduction of muscle power and to encourage normal motor development using a range of therapeutic skills		4	9
Newborns/Infants: prevention of new blisters is attempted by gentle handling		3	7
Care for individuals with EB requires a multidisciplinary team (MDT) for interdisciplinary care (IDC)		3	8
**R2. Optimize safe and functional mobility in their natural environment**	**C**		
Across all major subtypes of EB, there exists a cumulative risk to develop musculoskeletal contractures in areas other than hands and feet; risk increases with age		1-	10
Exercise the feet to keep them in good health		4	17
Aid of a lower extremity prosthesis* along with a cane, allows for community distance ambulation		3	23
Wearing a lower extremity prosthesis and not utilizing a cane, allowed for household ambulation		3	23
Mobility increases muscle strength, balance and coordination in RDEB patients		3	11
**R3. Optimize/Enhance/Elevate ambulation endurance and cardiorespiratory function**	**D**		
Mobility was a stronger predictor of bone mineral content than the type of EB		3	15
Promotion of gentle weight bearing activity such as standing and walking, including interventions such as vibratory platforms, maybe helpful		3	14
Provide mobility aids, such as tricycles, for safe longer distance use by the more severely affected patients		4	9
Mobility is positively associated with bone mineral density (BMD); increase in BMD is correlated with higher category of weight-bearing activity		4	13
Verified small body size and inactivity are associated with low BMD		3	13
Weight bearing activity and mobility suggested to be a significant factor in improving BMD in adults with RDEB		3	11
** R4. Optimize ability to safely bear weight**	**D**		
Full physiotherapy assessment of all joint ranges of movement, muscle power, gross motor abilities and motor development should be performed		4	9
For infants, knee padding and soft special shoes are required to prevent blistering		4	2, 26
Provide advice on footwear		4	9, 17 18
Orthotics/splints may also be necessary with careful choice of materials, can be useful adjunct to therapy		4	9
Semi-rigid orthotics are functional and cushioning; they provide support and allow movement, with a cushioning element and reduced friction		4	17
Use of a lower extremity prosthesis* after unrelated fractures, allowed return to weight bearing activities		3	23
**R5. Improve access to appropriate PT services**	**D**		
An early rehabilitation consult with frequent re-evaluations is recommended and essential for optimum management to encourage and facilitate weight-bearing activity and independent function		3, 4	9, 26, 21
In some areas, specialized EB Clinics exists with comprehensive care in one visit		3	7
As not all patients can be looked after in specialized centers, non-EB practitioners should seek support from established EB Centers, EB Care Network or ‘DEBRA’ foundations		3, 4	7, 26
Physiotherapists are recognized as an integral member of the multidisciplinary team (MDT)		3, 4	7, 19, 20
Education regarding role of physiotherapy		3, 4	19–21
The goal to optimize EB patient healthcare requires the implementation of a wide range of measures by establishing in each country a center of expertise. EB Centers would guarantee a multidisciplinary care service. Equally essential is the sharing of standards of care among expertise centers		4	20
B. The balance between desirable and undesirable consequences *is closely balanced or uncertain*, for this reason we suggest offering this option			
**R6. Optimize interaction with environment**	**D**		
Fostering independence and safety during activities of daily living (ADLs) requires environmental modifications (i.e.: wheelchair and footwear)		4	28
Proper fit of an upper extremity prosthesis* allowed continuation with most ADLs to achieve quality of life (QoL) and functional goals, such as driving and continuation of work		3	22
Lower leg prosthesis* for a generalized DEB person provided ability to achieve full weight bearing after leg amputation with and without a cane for community and home ambulation, respectively		4	23

**KEY**: R, recommendation; EB, epidermolysis bullosa; DEB, dystrophic epidermolysis bullosa; RDEB, Recessive dystrophic EB; DEBRA, Dystrophic Epidermolysis Bullosa Research Association; MDT, multidisciplinary team; IDC, interdisciplinary care; BMD, bone mineral density; ADLs, activities of daily living; QoL, quality of life.

*Definition: Prosthesis is defined as an artificial limb

Recommendation panel subgroup voting consensus

There was 100% agreement of panel members who attended meeting and when shared with complete panel after recommendations were written, there were no additional comments to change

**Levels of Evidence** [5]

4 = expert opinion; 3 = Non-analytic studies, e.g. case reports, case series; 1- = Meta analyses, systematic reviews of RCTs, or RCTs with a high risk of bias

**Grades for Strength of Recommendations** [6]

No A or B in table; present is one C = Extrapolated evidence from studies rated as 1- and all others were D = Evidence level 3 or 4; or Extrapolated evidence from studies rated as 2 +

**Recommendation 1**. We suggest consideration to optimize developmental motor milestone attainment *(Strength of Recommendation: D).*

Based on the evidence, we suggest that newborns and infants with EB receive developmental activities and positioning in order to optimize developmental motor milestone attainment from a physiotherapist. At birth and during prolonged hospitalizations, a physiotherapist, as a member of the IDT, can provide consultation for developmental activities and developmental positioning [7, 8]. Newborns and infants suspected or diagnosed with EB should be handled by gentle lifting through the ‘cradle carry’ technique to limit and prevent unnecessary shearing forces that could result in blistering. Infants should be lifted by placing one hand beneath the infant/child’s bottom and one hand behind the neck, rather than lifting from under the arms, to minimize friction and blister development in the trunk, axillary and arm areas [7]. Gross motor developmental milestones are an integral part of their physical growth. Daily prone lying is recommended for all infants with EB to help delay potential soft tissues shortening and adherence [9]. A physiotherapist can provide guidance for cautious placement of an infant into a modified and/or traditional prone position on soft surfaces, while reducing shearing forces. A physiotherapist should use standardized assessments and outcomes measures to identify baseline gross motor skills and to formulate intervention strategies if there are gross motor delays (Additional file 3). If there are no current gross motor delays observed, a physiotherapist can provide anticipatory guidelines for age appropriate gross motor developmental activities (Table 2).

### *Expert practice points*

- Identify soft surfaces, such as double lined fabric or sheep skin, when cradling or carrying an infant in order to decrease shearing forces.
- In addition to bandage wrapping, additional padding placed along the front of the knees and base of the palm can provide extra cushion to infants when they bear weight during crawling or creeping.

**Recommendation 2**. We suggest consideration to optimize safe and functional mobility in their natural environment *(Strength of Recommendation: C).*

For individuals with EB who are ambulatory and non-ambulatory, we suggest optimizing safe and functional mobility in their natural environment by providing mobility devices, enhancing functional activities, and endurance training through stretching, strengthening and balance exercises. Restrictions in joint and muscle movement due to blistering and repeated healing processes result in a shortened range of motion and can lead to permanent musculoskeletal abnormalities. These musculoskeletal abnormalities across the body would benefit from traditional physical therapy interventions, such as gait analysis, stretching exercises, strengthening activities, balance and coordination activities from clinicians who are familiar with EB [10, 11]. These exercises are taught to caregivers/parents and encouraged to be incorporated into daily life for functional and realistic application. A physiotherapist can provide direction on how to delay loss of functional independence prior to recommending use of durable medical equipment (DME) and continue to encourage regular activities for as long as possible. Identifying and incorporating personal and social purposes as one of the primary goals can facilitate individuals with EB to maintain mobility [11]. When the ability to independently interact with the community becomes compromised, begins to decline, or is impacted, a referral to a physiotherapist can be made to evaluate and assess the need for a mobility device. A physiotherapist can collaborate with a DME specialist(s) to identify the least restrictive mobility device for a patient’s current lifestyle with potential for growth modifications or for changes in function. When resources are limited, a physiotherapist can explain the purpose and provide examples of the least restrictive mobility devices to facilitate creative means out of local resources (Table 2).

### *Expert practice points*

- Discuss wrapping techniques to facilitate rather than limit functional mobility/proper movement
- When bandages cross the ankle joint, support a sub-talar joint neutral position and/or pull in direction of relative dorsiflexion to actively reduce the degree of plantarflexion contractures

**Recommendation 3**. We suggest consideration to optimize/enhance/elevate ambulation endurance and cardiorespiratory function *(Strength of Recommendation: D).*

For individuals with EB, a physiotherapist would educate on bone health, weight bearing activities, standing frames, balance and strengthening activities to optimize ambulation, endurance and cardiorespiratory function across an individual’s lifespan. Individuals with EB and significant contractures or palpable yet immeasurable tightness, should regularly participate in active exercises [9]. A physiotherapist can provide an individualized, progressive home exercise program that is incorporated into daily life on how to facilitate functional and endurance training activities as well as strengthen bones and muscles. Individuals with EB who are physically active usually have more autonomy and delayed wheelchair dependence [12]. Skin fragility, pain, joint contractures, and fatigue are some of many factors that contribute to immobility in this population [13]. Limited mobility has been shown to predict low bone mass in EB [14] and the level of mobility is a significant predictor of bone mineral content [15]. Patients with EB should be encouraged to maintain the highest level of weight-bearing activity that is tolerable [12, 13]. Another study showed that if RDEB individuals attained puberty, maintain mobility and optimize nutrition, it is possible to improve bone mass density while peak bone mass is being accrued [11]. Sporting activities have been recommended to strengthen muscles and bones and to improve overall QoL [12]. Continuing to maintain weight bearing activities as well as flexibility allows persons with EB to maintain and improve their cardiovascular function (Table 2).

### *Expert practice points*

- Educate school personnel on the importance of continued ambulation versus utilizing a wheelchair when navigating school settings.
- Educate adaptive physical education programs on the importance of continued ambulation versus utilizing wheelchair when navigating school settings.

**Recommendation 4**. We suggest consideration to optimize ability to safely bear weight *(Strength of Recommendation: D).*

We suggest to optimize the ability to safely bear weight, a physiotherapist will recommend biomechanically appropriate shoe attire and insole. Varying degrees of reported pain can affect functional mobility, ability to ambulate and footwear choices even in the mildest localized forms of EBS [16]. When blistering is provoked by use, the ability to walk can be significantly reduced [9]. A thorough physiotherapy evaluation is suggested to identify range of motion limitations, muscle power limits, skin integrity and trigger spots of friction to assess need for socks, orthotics and shoes. In collaboration with orthotists and/or podiatrists, suggestions for footwear can include items that will have as few internal seams as possible and will be made of soft material or be lined with sheepskin with a supportive sole to endure the natural environmental elements. In addition to shoes, the use of socks, such as silver-lined socks, that can conduct heat away from the feet, thereby cooling the feet and stopping the buildup of friction and pressure [17, 18]. Also, for individuals who do not wrap their lower extremity, wearing 2 pairs of thin breathable socks, with one worn on top of the other, can keep the layers of friction to a minimal amount within the shoe. This method helps to prevent friction between the skin and the shoes, especially when a person is walking or jogging [17]. Supportive orthotics to maintain foot and ankle alignment, along with proper footwear, will reduce shearing forces and friction leading to fewer blisters, allowing optimal ability to safely bear weight (Table 2).

### *Expert practice points*

- An individualized assessment is important to guide the recommendation of footwear.
- Discuss/share general characteristics of shoe types/footwear, sock material, and images of orthotics and prosthetics to allow local resources to be utilized in creating biomechanically appropriate footwear as well as engage the patient in their own personal preferences, as far as possible.

**Recommendation 5**. We suggest consideration to improve access to appropriate physiotherapy services *(Strength of Recommendation: D).*

By providing education regarding physiotherapy services to local healthcare providers, caregivers, parents and public, we suggest this will improve access to appropriate physiotherapy services. The complete care of patients with EB requires an IDT that includes physiotherapist as a core member [7, 11, 18–20]. Physical therapy must be started early in life, in particular in EBS with muscular dystrophy, generalized RDEB and JEB subtypes. The continuing work on muscles and joints delays contractures and deformities, improves functional mobility, enhances patient autonomy and ultimately ensures individuals with EB have a greater capacity for social integration with their peers. Static (preventive) and dynamic (corrective) orthosis, directed therapeutic exercises and recreational activities are empowering tools. Some forms of physical treatment, such as hydrotherapy, are also useful [20]. The IDT approach is often found in metropolitan areas and specialized EB centers. If such a center is not available, a dermatologist or pediatrician should coordinate with the various specialists via consultation. It is important to seek specialists who are familiar with EB. Knowledge sharing among physiotherapist to manage mobility [21] and reduce or avert musculoskeletal complications such as webbing and contractures would be beneficial to educate health care practitioners unfamiliar with EB or who practice in a rural area [19]. As EB is a rare diagnosis, a standard physiotherapy evaluation template is provided as a guideline (Additional file 4). In the case of JEB, 32% of providers recommended follow-up with a nutritionist or dietician with experience of EB every 3 to 6 months, whereas only 14% of providers recommended visits every 3 months with a physiotherapist or OT [19]. Prior to any hospital discharge, as well as depending on the country, the national and regional laws, there needs to be documentation that must address public health services to guarantee homecare by specialized services, including physiotherapist [20] (Table 2).

### *Expert practice points*

- Educate the national and local physiotherapy organizations and chapters about EB
- Physiotherapists to provide education to EB nurses at EB Centers and Wound Care Teams
- Knowledge share with DEBRA International CPG Network to collaborate with primary physicians (i.e.: Family medicine, pediatricians, and general dermatologists and subspecialists, such as orthopedic and gastroenterologist)
- Knowledge share information with National Organization of Rare Diseases (NORD)

**Recommendation 6**. We suggest to optimize interaction with environment *(Strength of Recommendation: D).*

To optimize interaction with their environment, a physiotherapist should provide intervention by assessing appropriate positioning and posture using lower extremity bracing and splinting, seating and mobility options for individuals with EB [25]. Physiotherapists evaluate and examine current movement patterns and body positioning within the environmental set up to assess and recommend the least restrictive and most supportive durable medical equipment, splints and/or orthotics to enhance interaction within the home and community. For individuals with DEB, physiotherapists collaborate with orthotists, prosthetists, podiatrists and vendors who specializes in durable medical equipment to improve their functional interaction within their environment [22]. Prosthesis for upper extremities can support continued activities of daily living to enhance QoL, such as driving to work [22]. For lower extremity prosthesis, the physiotherapist can focus on upright weight bearing therapeutic activities to improve ambulation within the home and community with or without the use of assistive devices [23]. When orthotics, prosthetics or durable medical equipment become ineffective at enriching or maintaining functional activities, a re-evaluation by a physiotherapist would be recommended to identify changes in the musculoskeletal system or cardiovascular system before addressing changes to the durable medical equipment (Table 2).

### *Expert practice points*

- Consider circumference of foot and ankle with bandages and/or layers of socks when recommending size of orthotics.
- During painful wound healing phases that limit functional mobility, physiotherapists advise utilizing a manual or power wheelchair to continue to optimize interactions within the environment.
- Tricycles for children and adults, as well as mobilized scooters, are another alternative to wheelchairs that can provide independence, strengthening, increased endurance and social interaction in the community.

The recommendation summary has been grouped by age if the recommendation refers to a specific age level, see Table 2. Infants are defined as birth to 12 months, children are defined as 1 year to pre-pubescent and adults are defined as post-pubescent. The majority of the articles were graded level 3, being small-scale case studies or level 4 for expert opinion, and with one article graded level 1. All recommendations were regarded as best practice based on the clinical experience of the guideline development group. Subtype specific recommendations are identified in Table 2 when the population sample is referenced by the evidence in the article.

## Conclusion

Individuals with EB can present to physiotherapy services with a wide range of deficits, including but not limited to joint contractures, strength and balance deficits, inability to bear weight, poor endurance, durable medical equipment needs, and pain that can lead to decreased functional interactions as an active participant in their family and within their community. Physiotherapy is an integral part of the IDT who evaluates and assesses gross motor developmental skills, impairments that limit functional movement within the home and community, weight bearing skills, durable medical equipment needs, endurance and education based on movement sciences. When not in active disease state, encourage physical and weight bearing activities to facilitate healthy bone mineral accrual [24]. This CPG provides evidence based recommendations to the physiotherapist who can safely address functional movement concerns with the EB community. It also outlines areas where more evidence is needed to improve physiotherapy management in EB (Table 3).

**Table 3:** Identifies deficits in Physiotherapy research by outcome

Current outcome	General areas for future research
Optimize developmental motor milestone attainment	Comparative studies regarding attainment of gross motor skills between infants with and without with EB Evaluate effectiveness of prone skill achievement between infants with and without EB Evaluate timeframe of typical developmental achievement for infants and toddlers with EB Studies to support early education/home exercise program for developmentally age appropriate gross motor milestones and handling from birth
Optimize safe and functional mobility in their natural environment	Longitudinal study to quantify functional gross motor skills through lifespan of persons with EB Long term retrospective study on effectiveness of an individualized physiotherapy home exercise program and functional mobility Long term prospective study regarding mobility devices and duration of maintaining independent functional mobility
Optimize ambulation endurance and cardiorespiratory function	Study to assess validity and reliability of Standardized Assessments and Outcomes Measures on people living with EB Evaluate effectiveness of a walking program on levels of endurance for people living with EB Assess whether use of tricycle/bicycle, rather than a wheelchair increase endurance and cardiorespiratory system lead to prolonged ambulation skills Identify cardiovascular endurance changes in persons with EB over a designated period of time Study cardiomyopathy and/or muscular dystrophy in persons with EB and exercise capacity Evaluate if techniques such as massage and/or compression garment would be effective in reducing edema
Optimize ability to safely bear weight	Long term study of lower extremity range of motion and direction of dressing application to prevent contractures Efficacy of supportive orthotics for range of motion and strength to bear weight for major types of EB
Optimize interaction with environment	Identify likely prognostic timeframe when durable medical equipment will be needed for the major types of EB Evaluate the efficacy of power wheelchair versus power scooter in the EB population
Improve access to appropriate physiotherapy services	Delphi research project incorporating the clinical expertise of international physiotherapists who care for persons with EB

CPGs in rare conditions have many challenges and limitations descending from small numbers of patient participation in studies to offer robust evidence. This results in a lack of good quality studies in therapies, such as physiotherapy in EB. This leads to a greater need for expert and clinical knowledge to be incorporated, but often there is limited and uneven distribution of knowledge, expertise and lack of EB-specializing professionals. In addition, our panel is predominately from pediatrics EB services as we found it rare for physiotherapists to specialize in adult EB services. 2020 also presented with the added difficulties of COVID 19 and reduced clinical capacity of many EB specialist physiotherapists, doctors and nurses, which did have an impact on getting more review panel members.

## Future research

Overall, the development of a physiotherapy management in EB handbook would be a beneficial tool with practical applications in the clinic setting. Table 3 details the possible specific research in areas where there is limited evidence.

## Updating and dissemination

It is anticipated that a literature search for new evidence pertaining to the provision of physiotherapy in EB will be undertaken every 3–5 years after the publication in order to update the guidelines. The CPG will be hosted by DEBRA international website (https://www.debra-international.org/eb-healthcare-resources) to ensure their availability and dissemination to clinicians, caregivers, and people with EB worldwide. DEBRA International recommends that the implementation of the CPG recommendations should be monitored and evaluated through audits. DEBRA International has designed a pre-implementation survey to help (https://surveyhero.com/c/aabc0100).

## Supplementary Information

**Additional file 1**: Panel roles and contribution and external review panel

**Additional file 2**: PICO, Key Terms and Search Engines

**Additional file 3**: Standardized Assessments and Outcome Measures

**Additional file 4**: Physiotherapy Evaluation Template for persons with EB

## Data Availability

Not applicable.

## Abbreviations

ADL: Activities of daily living
AGREE: Appraisal of Guidelines for Research and Evaluation
BMD: Bone mineral density
CASP: Critical Appraisal Skills Programme
CPG/CPGs: Clinical practice guideline(s)
DDEB: Dominant dystrophic EB
DME: Durable medical equipment
EB: Epidermolysis bullosa
EBS: EB simplex
DEB: Dystrophic EB
GI: Gastrointestinal
GRADE: Grades of Recommendation Assessment, Development and Evaluation
IDT: Interdisciplinary team
JEB: Junctional EB
KEB: Kindler EB
MDT: Multidisciplinary team
NORD: National Organization of Rare Diseases
OT/OTs: Occupational therapist(s)
QoL: Quality of life
RDEB: Recessive dystrophic EB
SIGN: Scottish Intercollegiate Guidelines Network
