# Supplementary material for: Physiotherapy for epidermolysis bullosa: clinical practice guidelines
Source: Orphanet J Rare Dis. 2021 Sep 30;16:406. doi: 10.1186/s13023-021-01997-w (PMC8481321; doi:10.1186/s13023-021-01997-w)
Supplement: Supplementary file 1 — Additional file 1: Panel roles and contribution and external review panel [file 13023_2021_1997_MOESM1_ESM.docx]

**Panel roles and contribution and external review panel**
