# Supplementary material for: Physiotherapy for epidermolysis bullosa: clinical practice guidelines
Source: Orphanet J Rare Dis. 2021 Sep 30;16:406. doi: 10.1186/s13023-021-01997-w (PMC8481321; doi:10.1186/s13023-021-01997-w)
Supplement: Supplementary file 2 — Additional file 2: PICO, Key Terms and Search Engines [file 13023_2021_1997_MOESM2_ESM.docx]

1. **PICOS breakdown**

| **Population** | **Intervention** | **Comparison** | **(Rank) Outcome** |
| --- | --- | --- | --- |
|  |  |  |  |
| Persons with EB: Pre-ambulatory | Developmental Activities and positioning | No PT treatment | (1) Optimize developmental motor milestone attainment |
| Persons with EB: Ambulatory and Non-ambulatory | Mobility devices, functional activities (stretching/ROM, balance, and strengthening exercises), endurance training |  | (2) Optimize safe and functional mobility in their natural environment |
|  | Encourage continued ability to ambulate by educating on bone health, weight bearing activities, stander/frames, balance and strengthening activities |  | (3) Optimize/Enhance/Elevate ambulation endurance and cardiorespiratory function |
|  | Recommend biomechanically appropriate shoe attire and insole |  | (4) Optimize ability to safely bear weight |
|  | Appropriate positioning and posture using Standing frames, LE bracing and splinting, soft cervical custom collar, seating and mobility options |  | (5) Optimize interaction with environment |
|  | Educating local healthcare providers, caregivers, parents, and public |  | (6) Improve access to appropriate PT services |

1. **Search Engines and Key Terms**

| **Search Engine** | Medline, PubMed, CINAHL, SCOPUS, PEDRO, ClinicalKey, EBSCO, Web of Science, Ovid | |
| --- | --- | --- |
| **Key terms** | **To be included in all:**  **Physiotherapy/Physical Therapy and Epidermolysis Bullosa**  Developmental Motor Milestones  Position(ing)  Weight bearing  Cervical soft collars  Cranial molding orthoses  Home exercise program  Education  Standardized assessments  Pain  Standers/Standing Frame  Range of motion  Mobility  Function | Strengthening  Stretching  Manual and Power wheelchair  Scooters  Gait Trainer  Balance  Endurance  Ambulation  Standardized assessments  Walkers  Empowerment  Community Resources  Lower extremity Orthoses,  Lower extremity braces, and splints  Shoes and socks  Cardiopulmonary/Cardiorespiratory Systems  Self-management |
