# Supplementary material for: Physiotherapy for epidermolysis bullosa: clinical practice guidelines
Source: Orphanet J Rare Dis. 2021 Sep 30;16:406. doi: 10.1186/s13023-021-01997-w (PMC8481321; doi:10.1186/s13023-021-01997-w)
Supplement: Supplementary file 3 — Additional file 3: Standardized Assessments and Outcome Measures [file 13023_2021_1997_MOESM3_ESM.docx]

**Standardized Assessments and Outcome Measures**

| Standardized Assessments and Outcome Measures | | |
| --- | --- | --- |
| Name | Description | Age Range |
| **The Alberta Infant Motor Scale (AIMS)** | Observational assessment scale constructed to measure gross motor maturation in infants from birth through independent walking. Motor skills are tested by observing the infant as they move into and out of four positions. Each item describes three aspects of motor performance: weight-bearing, posture, and antigravity movements. The AIMS provides information about the infant’s current motor function and may assist in identifying missing components of motor tasks and formulating intervention strategies [27-28]. | 0 -18 months |
| **Peabody Developmental Motor Scale- 2 (PDMS-2)** | The PDMS-2 measures interrelated motor abilities that develop early in life and is designed to assess the motor skills of children from birth through 5 years of age. The Gross Motor Quotient (GMQ) is a numeric representation of a child’s overall performance on three of four subtests (reflexes, stationary, locomotion, and object manipulation) that measure the use of the larger muscle systems. The following subtests form this composite score: Reflexes, Stationary, Locomotion and Object Manipulation [29]. | Birth - 5 years old |
| **Bruininks-Oseretsky Test of Motor Proficiency (BOT-2)** | The BOT-2 is a standardized, norm-referenced measure of fine and gross motor skills of children and youth, 4 through 21 years of age. For gross motor skills, only two sections are administered including body coordination with subsections of bilateral coordination and balance, and strength and agility with subsections of running speed and agility and strength [29]. | 4 years to 21 years old |
| **Dynamic Gait Index (DGI)** | Developed as a clinical tool to assess gait, balance and fall risk. It evaluates not only usual steady-state walking, but also walking during more challenging tasks. It assesses the individual’s ability to modify balance while walking in presence of external demands [30]. | All |
| **Pediatric Balance Scale** | Modified version of the Berg Balance Scale that is used to assess functional balance skills in school-aged children. The scale consists of 14 items that are scored from 0 points (lowest function) to 4 points (highest function) with a maximum score of 56 points [31]. | School Aged Children |

**References for this section:** 27. Darrah J, Piper M, Watt M-J. Assessment of gross motor skills of at-risk infants: predictive validity of the Alberta Infant Motor Scale. Dev Med Child Neurol. 1998; 40:485-491. 28. Song YH, Chang HJ, Shin YB, Park YS, Park YH, Cho ES. The validity of two neuromotor assessments for predicting motor performance at 12 months in preterm infants. Ann Rehabil Med. 2018; 42: 296-304. 29. Griffiths A, Toovey R, Morgan PE, et al. Psychometric properties of gross motor assessment tools for children: a systematic review BMJ Open. 2018;8. 30. Anne Shumway-Cook, Catherine S. Taylor, Patricia Noritake Matsuda, Michael T. Studer, Brady K. Whetten, Expanding the Scoring System for the Dynamic Gait Index, *Physical Therapy*. 2013; 93,1493–1506. 31. Franjoine, Mary, Rose PT, DPT, MS, PCS, et al. The Performance of Children Developing Typically on the Pediatric Balance Scale. PEDIATR. PHYS. THER.2010;22:350-359.
