# Supplementary material for: Physiotherapy for epidermolysis bullosa: clinical practice guidelines
Source: Orphanet J Rare Dis. 2021 Sep 30;16:406. doi: 10.1186/s13023-021-01997-w (PMC8481321; doi:10.1186/s13023-021-01997-w)
Supplement: Supplementary file 4 — Additional file 4: Physiotherapy Evaluation Template for persons with EB [file 13023_2021_1997_MOESM4_ESM.docx]

Physical Therapy Evaluation for Epidermolysis Bullosa

| **Patient/Client Information** | | | |
| --- | --- | --- | --- |
| **Name** |  | **Medical record/**  **Client ID** |  |
| **Date of Birth** |  | **Evaluation/**  **Service Date** |  |
| **Chronological Age** |  | **Number of Visits** |  |
| **Adjusted Age** |  | **Phone Number** |  |
| **Address** |  | **Mobile Number** |  |
| **Email** |  |  | |
| **Gender & Sex** |  | **Diagnosis code** | |

| **EB/Medical History** | | | |
| --- | --- | --- | --- |
| **Type of EB** |  | **Primary Care Provider (PCP)** |  |
| **Other Known Conditions or**  **Diagnoses** |  | **PCP Address** |  |
| **Medications** |  | **PCP Phone Number** |  |
| **Precautions** |  | **Patient/**  **Client ID** |  |
| **Dressing Materials** |  | | |
| **Insurance/Payer** |  | | |
| **Other Health Care Providers** |  | | |

| **Service Provider Information** | |
| --- | --- |
| **Service provided** |  |
| **Direct patient/**  **client care time (minutes)** |  |
| **Indirect patient/**  **client care time (minutes)** |  |
| **History of Current Presentation** |  |
| **Pertinent Medical History** |  |
| **Gross Motor/**  **Developmental or Functional History** |  |

| **Social History** | | | |
| --- | --- | --- | --- |
| **Family in Home** |  | **Primary Provider of Assistance in Home** |  |
| **Relationship Status** |  | **Type of Home** |  |
| **Caregiver Roles** |  | **Stairs In/At Home** |  |
| **Home environment** |  | | |
| **School** |  | | |
| **Work** |  | | |
| **Therapies received** |  | | |
| **Equipment** |  | | |

| **Primary Concerns** | |
| --- | --- |
| **Patient** |  |
| **Caregiver** |  |

| **Pain** | |
| --- | --- |
| **Pain at Best** |  |
| **Pain at Worst** |  |
| **Aggravating Factors** |  |
| **Easing Factors** |  |
| **Nature of Pain** |  |
| **24-hour pain behavior** |  |

| **Functional Limitations** |
| --- |
|  |
| **General Observations** |
|  |

| **Skin Integrity** | |
| --- | --- |
| **Hands** |  |
| **Upper extremity** |  |
| **Feet** |  |
| **Lower extremity** |  |
| **Neck** |  |
| **Trunk** |  |

| **Posture** | | | |
| --- | --- | --- | --- |
| **Sitting** |  | **Standing** |  |

| **Range of Motion (Active/Active Assist/Passive)** |
| --- |
|  |

| **Strength** | | | |
| --- | --- | --- | --- |
|  | | | |
| **Leg Length** | | | |
|  | | | |
| **Palpation** | | | |
|  | | | |
| **Neurological Screening** | | | |
| **Tone** |  | **Spasticity** |  |

| **Sensation Screening** | |
| --- | --- |
| **Tactile** |  |
| **Light touch**  **(dorsal column)** |  |
| **Vibration**  **(dorsal column)** |  |
| **Deep Pain (anterolateral)** |  |
| **Pin Prick (anterolateral)** |  |
| **Thermal Sense (anterolateral)** |  |
| **Proprioception** |  |
| **Kinesthesia** |  |
| **Vestibular** |  |
| **Auditory** |  |
| **Visual** |  |

| **Functional Movement Analysis** | |
| --- | --- |
| **Bed Mobility** |  |
| **Gait** |  |
| **Running** |  |
| **Stairs** |  |
| **Squats** |  |
| **Jumping/Hopping** |  |

| **Developmental Assessment** | |
| --- | --- |
| **Transitions** |  |
| **Supine** |  |
| **Prone** |  |
| **Sitting** |  |
| **Quadruped/Creeping** |  |
| **Standing** |  |
| **Ambulation** |  |
| **Stairs** |  |

| **Balance** | |
| --- | --- |
| **Static Sitting** |  |
| **Static Standing** |  |
| **Dynamic Sitting** |  |
| **Dynamic Standing** |  |
| **Single Limb Stance** |  |
| **Rhomberg** |  |
| **Tandem Walking** |  |
| **Functional Reach** |  |

| **Coordination** | |
| --- | --- |
| **Finger to nose** |  |
| **Heel to Shin** |  |
| **Rapid Alternating** |  |
| **Movements** |  |
| **Fingertip Touching** |  |
| **Braiding** |  |
| **Sidestepping** |  |
| **Walk on Toes** |  |

| **Endurance** |
| --- |
|  |
| **Standardized Tests** |
|  |

| **Home Exercise Program/Caregiver Education** |
| --- |
| **Was provided with a home program including:** |
| **Assessment** |
|  |

| **Recommendations/Plan** | |
| --- | --- |
|  | |
| **Goals** |  |
|  |  |
| **FOLLOW UP/PLAN OF CARE** |  |
|  |  |
